# Retraction Note: Triplanar osteotomy combined with proximal tibial transverse transport to accelerate healing of recalcitrant diabetic foot ulcers

**DOI:** 10.1186/s13018-023-04351-x

**Published:** 2023-11-17

**Authors:** Jia Xu, Shanyu Li, Yunchu Sun, Bingbo Bao, Tianhao Zhu, Qinglin Kang, Xianyou Zheng, Gen Wen

**Affiliations:** Department of Orthopedic Surgery, Shanghai Jiao Tong University Affiliated Sixth People’s Hospital, 600 Yishan Road, Xuhui District, Shanghai, 200233 China

**Retraction Note : Journal of Orthopaedic Surgery and Research (2022) 17:528** **https://doi.org/10.1186/s13018-022-03410-z**

The Editor-in-Chief has retracted this article. Following publication, concerns were raised that the study reported in this article included data from patients who had undergone tibial transverse transport (TTT) as early as 2015. The authors were asked to provide example scans from 2015 from their dataset. However, the scans provided did not depict the required areas and the procedures discussed in the article. The Editor-in-Chief therefore no longer has confidence in the results and conclusions presented. None of the authors has responded to correspondence from the Publisher about this retraction.

